# Prevalence of *IGFBP3, NOS3* and *TCF7L2* polymorphisms and their association with hypertension: a population-based study with Brazilian women of African descent

**DOI:** 10.1186/s13104-021-05598-5

**Published:** 2021-05-17

**Authors:** Abel Barbosa Lira Neto, Nancy Borges Rodrigues Vasconcelos, Tamara Rodrigues dos Santos, Luisa Elvira Cavazzani Duarte, Monica Lopes Assunção, Carolinne de Sales-Marques, Haroldo da Silva Ferreira

**Affiliations:** 1 Federal University of Alagoas, Institute of Biological and Health Sciences, Postgraduate Program in Health Sciences, Campus A.C. Simões, Highway BR 104 North, Tabuleiro Do Martins, Maceió, Alagoas 57072-970 Brazil; 2 Rua Costa Gama, 1160, Caçimbas, Arapiraca, Alagoas 57038-430 Brazil

**Keywords:** Nitric oxide synthase, Hypertension, IGFBP3 human protein, Oxidative stress, African Continental Ancestry Group

## Abstract

**Objective:**

African ancestry seems to be a risk factor for hypertension; however, few genetic studies have addressed this issue. This study aimed to investigate the prevalence of polymorphisms *NOS3; rs1799983*, *IGFBP3; rs11977526* and *TCF7L2*; *rs7903146* in Brazilian women of African descent and their association with hypertension.

**Results:**

The prevalences of the less frequent genotypes were 26.5% TT genotype of *NOS3*; *rs1799983*, 16.7% AA genotype of *IGFBP3; rs11977526*, and 18.3% TT genotype of *TCF7L2; rs7903146*. For these conditions, the prevalence of hypertension and PR (adjusted) relatively to the ancestral genotype were, respectively: 52.0% vs 24.5% (PR = 1.54; *p* < 0.001), 62.0% vs 24.1% (PR = 1.59; *p* < 0.001), and 38.9% vs 27.9% (PR = 0.86; *p* = 0.166). Associations with hypertension were statistically significant, except for the *TCF7L2*; *rs7903146* polymorphism, after adjusted analysis. Brazilian Afro-descendant women with the TT genotype for the *NOS3* gene and the AA genotype for the *IGFBP3* gene are more susceptible to hypertension. The understanding of underlying mechanisms involving the pathogenesis of hypertension can motivate research for the development of new therapeutic targets related to nitric oxide metabolism and the management of oxidative stress.

**Supplementary Information:**

The online version contains supplementary material available at 10.1186/s13104-021-05598-5.

## Objective /Introduction

Considered a disease of multifactorial etiology, hypertension is more prevalent among people of African descent [1]. Although there is a higher prevalence of hypertension in Afro-descendant populations in comparison with other ethnicities, studies involving the association of single nucleotide polymorphism (SNP) with this pathology have mostly been conducted with people of European ancestry, and few studies are dealing with populations of African origin [2, 3].

The endothelial dysfunction of hypertension is mainly characterized by a non-relaxation of blood vessels caused by lower bioactivity of nitric oxide (NO) in the vascular wall, due to oxidative stress, causing an imbalance between the antioxidant and pro-oxidant systems, and leading to the prevalence of deleterious actions of reactive oxygen species on cells, tissues and organs [4, 5].

*IGFBP3* is a protein with the function of regulating the bioavailability of IGF-1 [6]. In vitro experiments indicate that IGFBP3 regulates IGF-1 by reducing vascular resistance when stimulating the synthesis of **NO** in endothelial cells [7]. Therefore, IGFBP3 serum levels are closely related to the production of endothelial **NO** and, consequently, to oxidative stress and hypertension [8].

Sedentary lifestyle, visceral adiposity, and insulin resistance are important risk factors for both hypertension and diabetes mellitus (DM). However, although several studies have demonstrated a relationship between the *TCF7L2* gene and DM, its relationship with the prevalence of arterial hypertension and oxidative stress has not been investigated [9, 10].

The SNPs in the *NOS3 rs1799983*, *IGFBP3 rs11977526*, and *TCF7L2 rs7903146* genes can directly influence the protein expression in the respective genes, making them important biomarkers for the development of hypertension. However, no studies addressing this association in Afro-descendant populations were found [11, 12].

Given the above, this study aimed to verify the prevalence of SNPs in the *NOS3 rs1799983*, *IGFBP3 rs11977526*, and *TCF7L2 rs7903146* genes, as well as to investigate the possible association of SNPs occurrence with arterial hypertension in Afro-descendant women, in quilombola communities in the state of Alagoas, northeastern Brazil.

## Main text

### Materials and methods

This is a household cross-sectional population-based survey, whose data were collected in remaining quilombola communities, in the state of Alagoas, Brazil. In the sample size calculation, hypertension was the was the variable of interest, whose prevalence in women of African descent was estimated at 35.8% [13]. The calculations were performed using the StatCalc software (Epi Info, version 3.5.4). For a sampling error of 3.0%, a 95% confidence interval, and adding 10% (852 + 85), to compensate for possible sample losses, 937 women would be needed.

Hypertension was the dependent variable, defined by systolic blood pressure (SBP) ≥ 140 mmHg and/or diastolic blood pressure (DBP) ≥ 90 mmHg and/or when the participant reported regular use of antihypertensive drugs [14].

The *NOS3; rs1799983*, *IGFBP3; rs11977526* and *TCF7L2*; *rs7903146* polymorphisms were the independent variables. For DNA extraction and polymorphisms testing, cell samples were collected from the women’s oral mucosa. The samples were stored in a refrigerator for subsequent DNA extraction using the salting-out method [15].

The *NOS3,* rs1799983, *IGFBP3,* rs11977526, and *TCF7L2*, rs7903146 SNPs were chosen for this study after bibliographic research in a complete genome database (Genome-Wide Association Studies—GWAS) [16, 17]. Genotyping was performed using the Step One Plus™ Real-Time PCR System (Applied Biosystems, Foster City, CA, USA), based on a previously standardized protocol [15].

The following covariables were used to control possible confounding factors and characterize the sample:

Demographic variables: age (19–30, 30.1–40, and 40.1–59 years).

Socioeconomic variables: unemployment (yes or no); per capita family income (≥ 1 minimum wage and < 1 minimum wage); *“Bolsa Família” Program* (yes or no); single register for social programs (yes or no); schooling level (≤ 4 years, > 4 years); self-reported race/skin color (Black/Brown; others: white, yellow or indigenous). Although the investigated population belongs to remaining quilombo communities (scenarios that, at the time of slavery in Brazil, were used as a refuge for fugitive African slaves), due to the miscegenation process, there are also people of other races/colors, although to a lesser extent than that of blacks and browns.

The food and nutrition security (FNS) or food and nutrition insecurity (FNI) was measured based on the Brazilian Food Insecurity Scale (EBIA) [18, 19].

Variables related to health and lifestyle: Alcoholism (yes or no); smoking (yes or no); physical activity level (PAL) measured based on the results obtained by applying the International Physical Activity Questionnaire (IPAQ) [20].

Anthropometric indicators: Body mass index (BMI; kg/m^2^) and waist circumference (WC) were used. The cut-off point proposed by the World Health Organization were used, obtaining the following categories: eutrophy (18.5–24.9 kg/m^2^), overweight (25.0–29.9 kg/m^2^), and obesity (≥ 30.0 kg/m^2^). The WC was measured with the woman standing. A cut-off point ≥ 80 cm was used to identify high cardiovascular risk or metabolic complications associated with obesity [21].

Biochemical variables: Total cholesterol and fractions, triglycerides, and diabetes mellitus were determined without mandatory fasting. The lipid profile was determined in an Alere Cholestech LDX® System (Abbott, USA). HbA1c was determined using a NycoCard Reader II® device (Abbott, USA) [22].

Data processing and analysis: Double independent data entry was performed using the Epi-Info^tm^ 3.5.4 software. The obtained database was exported to the Stata/SE 12.1 software for Windows (StataCorp LP, College Station, TX, USA), through which all analyses were performed.

According to the chi square test, all genotypes behaved according to the balance as Hardy–Weinberg equilibrium (HWE) [23].

The distribution adherence to parametric assumptions was verified using the Kolmogorov–Smirnov test. Thus, the means were subjected to analysis of variance (ANOVA), and the medians were tested using the Kruskal–Wallis test. Bonferroni and Dunnet post-hoc tests were used, respectively.

**Fig. 1:**
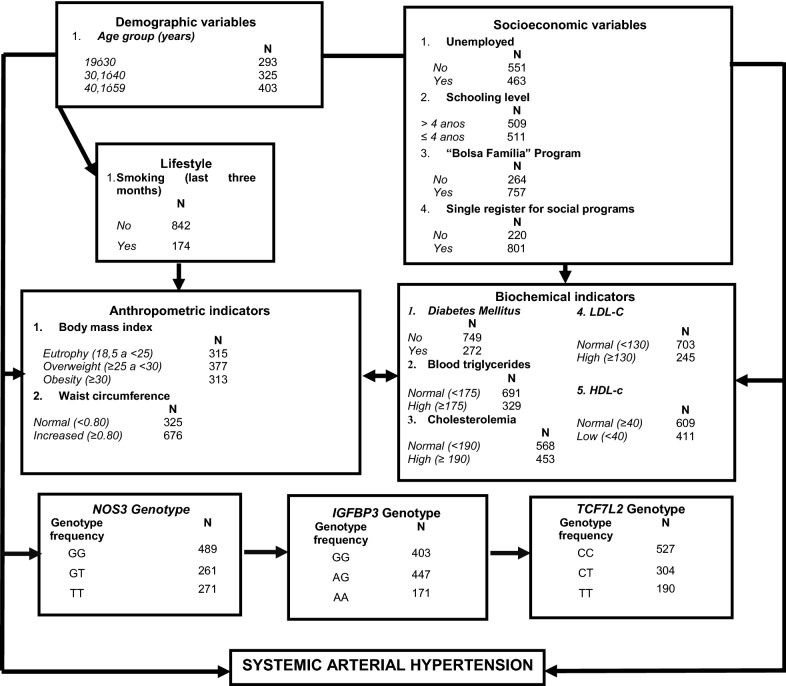
Hierarchical conceptual model explaining systemic arterial hypertension ( Adapted from Neto et al. [15])

Multiple analysis was performed according to an adapted four levels hierarchical theoretical model. (NETO et al. [15]) (Fig. 1). To identify an association between hypertension and the polymorphism genotypes, prevalence ratio (PR), and respective confidence interval (95% CI), were used, which were calculated using Poisson regression with robust variance.

## Results

The sample was composed of 1021 women (37.9 ± 10.9 years old), most of them self-declared as African/hispanic (91.1%). The prevalence of hypertension among them was 31.4% the sample characterization according to socioeconomic, demographic, lifestyle, anthropometric, biochemical, and genetic variables are shown in Additional file 1: Table S1.

The prevalences of genotypes for *NOS3*; rs1799983 SNP were GG = 47.9%, GT = 25.6% and TT = 26.5%, which was the least frequent genotype. The prevalences for *TCF7L2;* rs7903146 SNP was CC = 51.6%, CT = 29.8% and TT = 18.6%. The *IGFBP3;* rs11977526 SNP had the following prevalences: GG = 39.5%, GA = 43.8% and AA = 16.7%. There was a statistically significant difference between blood pressure levels and the *NOS3,* rs1799983 and *IGFBP3* rs11977526, SNPs, with emphasis on the genotypes: TT *NOS3*; rs1799983, SNP and AA *IGFBP3;* rs11977526 SNP.

The less frequent genotypes of the *NOS3,* rs1799983, *TCF7L2* rs7903146, and *IGFBP3* rs11977526, SNPs were associated with a higher prevalence of hypertension in comparison with the ancestral and heterozygous genotypes. The distribution of polymorphisms in accordance with the Hardy–Weinberg equilibrium (Additional file 1: Table S2).

Results similar to those recorded for distribution of prevalence were found when analyzing the measures of central tendency (mean and median) related to systolic blood pressure (Table 2). The values found for genotypes of *NOS3,* rs1799983 and *IGFBP3* rs11977526 were significantly higher than those obtained for the other genes, both in the ANOVA, in the Kruskal–Wallis nonparametric test. For the *TCF7L2,* rs7903146 SNP, PAS levels were considered statistically similar (*p* > 0.05 in both analyses).

For the *NOS3,* rs1799983 SNP, the prevalences of hypertension among women with GG, GT and TT genotypes were 24.5%, 23.0% and 52.0%, respectively, with a statistically significant difference between TT and GG (PR = 1.54; 95% CI 1.27–1.86; *p* < 0.001). Regarding *IGFBP3;* rs11977526 SNP, the prevalences of hypertension among women with GG, GA and AA genotypes were 24.1%, 26.4% and 62.0%, respectively, with a statistically significant difference between AA and GG (PR = 1.59; 95% CI 1.27–1.98; *p* < 0.001). For *TCF7L2;* rs7903146 SNP, hypertension among women with CC, CT and TT genotypes had prevalences of 27.9%, 32.9% and 38.9%, respectively, with no statistical difference between TT and CC (PR = 0.86; 95% CI 0.69–1.06; *p* = 0.166) (Table 1).

**Table 1:** Prevalence ratios (PR) and respective 95% confidence intervals (95% CI) obtained by multivariable Poisson regression, according to the hierarchical theoretical model for determining arterial hypertension

Variables	Level 1 PR (95% CI)	P	Level 2 *PR (95% CI)	P	Level 3 *PR (95% CI)	P	Level 4 PR (95% CI)	P
Level 1								
Age group: *30.1–40*	3.95 (2.46–6.34)	< 0.001	3.95 (2.46–6.34)	< 0.001	3.54 (2.20–5.69)	< 0.01	3.36 (2.10–5.36)	< 0.01
Age group: *40.1–50*	8.06 (5.16–12.60)	< 0.001	8.06 (5.16–12.60)	< 0.001	7.33 (4.69–11.48)	< 0.01	6.78 (4.35–10.59)	< 0.01
*Insertion in the formal labor market*	1.18 (0.99–1.41)	0.050	1.18 (0.99–1.41)	0.051	1.17 (0.99–1.40)	0.06	*	*
*Schooling level:* ≤ *4 years*	1.21 ( 0.99–1.48)	0.060	1.21 ( 0.99–1.48)	0.056	*	*	*	*
*“Bolsa Família” Program: yes*	1.20 (1.01–1.41)	0.037	1.20 (1.01–1.41)	0.037	1.15 (0.98–1.36)	0.094	1.12 (0.96–1.31)	0.156
*Single register for social programs: yes*	1.06 (0.78–1.44)	0.680	*	*	*	*	*	*
Level 2								
Smoking	–	–	0.99 (0.77–1.13)	0.48	*	*	*	*
Level 3								
*BMI Overweight (*≥ *25–* < *30)*					1.26 (0.98–1.61)	0.067	1.29 (1.02–1.64)	0.033
*BMI Obesity (*≥ *30)*	–	–	–	–	1.63 (1.29–2.06)	< 0.001	1.63 (1.29–2.06)	< 0.001
Waist circumference ≥ 80 cm	–	–	–	–	1.02 (0.72–1.43)	0.091		*
Triglycerides ≥ 175 mg/dL	–	–	–	–	1.05 (0.88–1.26)	0.551	*	*
Total cholesterol ≥ 190 mg/dL	–	–	–	–	1.20 (0.96–1.49)	0.101	*	*
*LDL-C (mg/dL) ≥ 160 mg/dL	–	–	–	–	0.90 ( 0.73–1.12)	0.384	*	*
*HDL-C (mg/dL) > *50 mg/dL*	–	–	–	–	1.27 (1.08–1.50)	0.003	1.24 (1.06–1.45)	0.007
Diabetes mellitus	–	–	–	–	1.28 (1.09–1.51)	0.003	1.16 (0.99–1.37)	0.060
Level 4								
*TCF7L2*								
CC	–	–	–	–	–	–	1	–
CT	–	–	–	–	–	–	1.04 (0.78–1.19)	0.623
TT	–	–	–	–	–	–	0.86 (0.69–1.06)	0.166
*NOS3*								
GG	–	–	–	–	–	–	1	–
GT	–	–	–	–	–	–	0.95 (0.74–1.23)	0.755
TT	–	–	–	–	–	–	1.54 (1.27–1.86)	< 0.001
*IGFBP3*								
GG	–	–	–	–	–	–	1	–
AG	–	–	–	–	–	–	0.96 (0.78–1.19)	0.751
AA	–	–	–	–	–	–	1.59 (1.27–1.98)	< 0.001

LDL, low-density lipoprotein; HDL, high density lipoprotein; Prevalence ratios and respective 95% confidence intervals (95% CI)

After the adjusted analysis *according to four levels hierarchical conceptual model*, older age group, family beneficiary of the “Bolsa Família” Program, obesity (BMI ≥ 30 kg/m^2^), low HDL-C level, and diabetes mellitus were significantly associated with hypertension.

The dominant and recessive model analysis was performed using the following risk factors: age group (30.1–40.0), age group (40.1–59.0), “Bolsa Família” Program, overweight (BMI ≥ 25), HDL-C, and glycated hemoglobin. In the dominant model of oxidative stress (*NOS3*: GG + *IGFBP3*: GG vs *NOS3* GT + TT; *IGFBP3*: AG + AA), women had a prevalence of 34.7% hypertension (PR = 1.42; 95% CI 1.12–1.79; *p* = 0.003). However, in the recessive model of oxidative stress (*NOS3*: TT + *IGFBP3*: AA vs *NOS3*: GG + GT *IGFBP3*: GG + AG), they had a prevalence of 77.0% hypertension (PR = 2.07; 95% CI1.78–2.42 *p* < 0.001). There was a statistical significance even after adjusting for all risk factors, in the dominant and recessive models, referring to the *NOS3* and *IGFBP3* genes (Table 2).

**Table 2:** Dominant and recessive models for determining systemic arterial hypertension in Brazilian women of African descent

Models	Hypertension (%)	*PR (95% CI)	P
*Dominant*			
*(NOS3 GG)* + *(IGFBP3 GG)*	21.3	1	–
*(NOS3 TT* + *GT)* + *(IGFBP3 AG* + *AA)*	34.7	1.42 (1.12–1.79)	0.003
*Recessive*			
*(NOS3 GG* + *GT)* + *( IGFBP3 GG* + *AG)*	26.5	1	–
*(NOS3 TT)* + *(IGFBP3 AA)*	77.0	2.07 (1.78–2.42)	< 0.001

Prevalence ratios (PR) and respective 95% confidence intervals (95% CI) adjusted by Poisson regression using the risk factors: age group (30.1–40.0), age group (40.1–59, 0), income supplementation program, overweight (BMI ≥ 25– < 30), obesity (BMI ≥ 30), HDL-C and diabetes mellitus; *hypertension: systemic arterial hypertension

## Discussion

This study provides evidence that, in Afro-descendant women from northeastern Brazil, the *NOS3,* rs1799983 and *IGFBP3,* rs11977526, SNPs gene are associated with higher blood pressure levels and, consequently with a higher prevalence of arterial hypertension, *except for the TCF7L2; rs7903146 polymorphism.*

A study in an African population investigated whether biomarkers of endothelial function were related to the bioavailability of IGF-1 (IGF-1, IGFBP3*,* or IGF-1/IGFBP3M ratio) and showed that the bioavailable IGF-1, measured by the IGF1/IGFBP3 ratio, is beneficially associated with CAV-1, which is a biomarker of endothelial activation [24]. Also, bioavailable IGF-1 tended to be inversely associated with ICAM-1, another marker of endothelial activation, thereby increasing the expression of CAV-1 and ICAM-1 [8, 25].

Previous studies indicated that some SNPs in the *IGFBP3* and *NOS3* genes are associated with decreased serum levels of these proteins [26–28]. Research on SNPs of the *IGFBP3* rs11977526 gene and hypertension indicated an association of these factors, as found in a study involving East African people, was associated with the risk of such disease [29, 30].

In this study, there was a higher predisposition for hypertension in the presence of TT and AA genotypes for the *NOS3* and *IGFBP3* genes, respectively. In the case of heterozygous Afro-descendant women (GT of the *NOS3* gene and AG of the *IGFBP3* gene), no statistically significant difference was found between the prevalence of hypertension. However, when analyzing the dominant and recessive models for oxidative stress, even after adjusting the risk factors, a significance was found for *NOS3* and *IGFBP3* models. Therefore, these data show that the presence of the TT genotype of the *NOS3* gene and the AA genotype of the *IGFBP3* gene constitutes an important risk factor for arterial hypertension.

The SNP rs1799983, variant of the *NOS3* gene causes a change in which the amino acid Asp is replaced by Glu at position 298. This substitution is associated with a decrease in protein stability [12, 31]. The AA genotype of the *IGFBP3* gene has been shown to regulate protein expression through miRNAs by destabilizing the mRNA, which is associated with a decrease in IGFBP3 serum levels [30, 32, 33].

In our analysis, the dominant and recessive showed a strong association of TT and AA genotypes of the *NOS3* and *IGFBP3* genes, respectively, with hypertension. CAV-1 is the main link between the *NOS3* gene and *IGFBP3* because it physically interacts with these gene regions, making possible a co-expression between the two proteins [34–36]. Recently, some studies have shown that CAV-1, which is a protein responsible for regulating eNOS function, is closely linked to IGF-1 and IGFBP3, regulating endothelial cell proliferation, vascular development, and oxidative stress [37–42].

## Conclusion

The TT genotype of the *NOS3* rs1799983 gene and the AA genotype of the *IGFBP3* rs11977526 gene are associated with a higher prevalence of arterial hypertension, *except for the TCF7L2; rs7903146 polymorphism, after adjusted analysis.* Considering that the mechanism of action, responsible for higher blood pressure levels in women with the TT (*NOS3*) and AA (*IGFBP3*) genotypes, involves less metabolic production of **NO** and, consequently, an increase in oxidative stress, the results presented here suggest that these SNPs are directly related to blood pressure regulation.

Future molecular studies are needed to reveal the important roles of eNOS and IGFBP3 when they are related to hypertension. Association studies such as the one presented here are of great relevance for motivating research aimed to elucidate the molecular pathways involved in the etiology of hypertension and, consequently, in the development of new drugs related to these pathways.

## Limitations

Thus, it did not provide for the inclusion of men and elderly people. Therefore, the absence of men is a limitation of this research. Due to the differences in the occurrence of hypertension by gender, further studies including male participants should be conducted.

## Supplementary Information

**Additional file 1: Table S1.** Distribution of systemic arterial hypertension the according to socioeconomic, demographic, lifestyle, anthropometric, and biochemical variables in women of African descent in the state of Alagoas (n = 1021). **Table S2.** Prevalence of arterial hypertension (%) and systolic blood pressure (mean ± SD; median and interquartile range), according to the genotypic frequencies for *NOS3 rs1799983, TCF7L2 rs7903146,* and *IGFBP3* rs11977526 genes. Brazilian women descended from African descent, 2018.

## Data Availability

The datasets used and/or analyzed during the current study are available from the corresponding author on reasonable request.

## Abbreviations

Asp: Aspartic acid
BMI: Body mass index
CI: Confidence interval
CAV-1: Caveolin-1
DNA: Deoxyribonucleic acid
DM: Diabetes mellitus
DBP: Diastolic blood pressure
EBIA: Brazilian Food Insecurity Scale
eNOS: Nitric oxide synthase
FNS: Food and nutrition security
FNI: Food and nutrition insecurity
Glu: Glutamic acid
GWAS: Genome-Wide Association Studies
HbA1c: Glycated hemoglobin
HDL: High density lipoprotei
HWE: Hardy–Weinberg equilibrium
ICAM-1: Intercellular Adhesion Molecule 1
IGF-1: Insulin-like growth factor-1
IGFBP3: Insulin-like growth factor binding protein -3
IPAQ: Physical Activity Questionnaire
LDL: Low-density lipoprotein
NO: Nitric oxide
PAL: Physical activity level
PR: Prevalence ratio
SBP: Systolic blood pressure
SNP: Single nucleotide polymorphism
WC: Waist circumference
