# Supplementary material for: Prevalence of IGFBP3, NOS3 and TCF7L2 polymorphisms and their association with hypertension: a population-based study with Brazilian women of African descent
Source: BMC Res Notes. 2021 May 17;14:186. doi: 10.1186/s13104-021-05598-5 (PMC8130172; doi:10.1186/s13104-021-05598-5)
Supplement: Supplementary file 1 — Additional file 1: Table S1. Distribution of systemic arterial hypertension the according to socioeconomic, demographic, lifestyle, anthropometric, and biochemical variables in women of African descent in the state of Alagoas (n = 1021). Table S2. Prevalence of arterial hypertension (%) and systolic blood pressure (mean ± SD; median and interquartile range), according to the genotypic frequencies for NOS3 rs1799983, TCF7L2 rs7903146, and IGFBP3 rs11977526 genes. Brazilian women descended from African descent, 2018. [file 13104_2021_5598_MOESM1_ESM.docx]

**Table -S****1** - Distribution of systemic arterial hypertension the according to socioeconomic, demographic, lifestyle, anthropometric, and biochemical variables in women of African descent in the state of Alagoas (n=1021).

| **Variables** | **No. (%)** | Hypertension **(%)** | ***P-value** |
| --- | --- | --- | --- |
| ***Age group (years)*** |  | ~~-~~ | ~~-~~ |
| *19–30* | 293 (28.7) | 6.5 | - |
| *30,1–40* | 325 (31.8) | 25.5 | <0.001 |
| *40,1–59* | 403(39.5) | 54.3 | <0.001 |
| ***“Bolsa Família” Program*** |  | - | - |
| *Yes* | 757 (74.1) | 27.2 | - |
| *No* | 264 (25.9) | 43.6 | <0.001 |
| ***Unemployed*** |  | - | - |
| *No* | 551 (54.3) | 34.6 | 0.017 |
| *Yes* | 463 (45.7) | 27.7 | - |
| ***Per capita income*** |  | - | - |
| *≥ Minimum wage* | 276 (34.0) | 31.5 | - |
| *< Minimum wage* | 536 (66.0) | 33.3 | 0.602 |
| ***Single register for social programs*** |  | - | - |
| *Yes* | 801 (78.4) | 28.3 | - |
| *No* | 220 (21.6) | 42.7 | <0.001 |
| ***Schooling level*** |  | - | - |
| *> 4 anos* | 509 (49.9) | 18.9 | - |
| *≤ 4 anos* | 511 (50.1) | 43.8 | <0.001 |
| ***Skin color (self-reported)*** |  | - | - |
| *Others* | 91 (8.9) | 28.6 | - |
| *African/Hispanic* | 928 ( 91.1) | 31.8 | 0.528 |
| ***Food Insecurity*** |  | - | - |
| *No (0)* | 266 (26.3) | 32.1 | - |
| *Yes (≥ 1)* | *746* (73.7) | 31.2 | 0.657 |
| ***Alcoholism*** |  | - | - |
| *No (0)* | 961 (94.6) | 31.4 | - |
| *Yes (≥1)* | 55 (5.4) | 32.7 | 0.840 |
| ***Smoking (last three months)*** |  | - | - |
| *No (0)* | 842 (82.9) | 29.5 | - |
| *Yes (≥1)* | 174 (17.1) | 41.4 | <0.002 |
| ***Physical activity level*** |  | - | - |
| *Active* | 394 (38.8) | 31.5 | - |
| *Sedentary* | 622 (61.2) | 31.9 | 0.83 |
| ***BMI classification (kg/m^2^)*** |  | - | - |
| *Eutrophy (18,5 a <25)* | 315 (31.3) | 19.6 | - |
| *Overweight (≥25 a <30)* | 377 (37.5) | 32.6 | <0.001 |
| *Obesity (≥30)* | 313 (31.1) | 42.5 | <0.001 |
| ***Waist circumference*** |  | *-* | - |
| *Normal (<0.80)* | 325 (32.5) | 17.2 | *-* |
| *Increased (≥0.80)* | 676 (67.5) | 38.8 | *<0.001* |
| ***Diabetes Mellitus*** |  | - | - |
| *No (0)* | 749 (73.4) | 26.0 | - |
| *Yes (≥1)* | 272 (26.6) | 46.3 | <0.001 |
| ***Triglycerides (mg/dL)*** |  | - | - |
| *Normal (<175)* | 691 (67.8) | 24.8 | - |
| *High (≥175)* | 329 (32.3) | 45.6 | <0.001 |
| ***Total cholesterol (mg/dL)*** |  | - | - |
| *Normal (<190)* | 568 (55.6) | 23.9 | - |
| *High (≥ 190)* | 453 (44.4) | 40.8 | <0.001 |
| ****LDL-C (mg/dL)*** |  | - | - |
| *Normal (<130)* | 703 (74.2) | 29.5 | - |
| *High (≥130)* | 245 (25.9) | 38.8 | <0.007 |
| ****HDL-C (mg/dL)*** |  | - | - |
| *Normal (≥40)* | 609 (59.7) | 26.6 | - |
| *Low (<40)* | 411 (40.3) | 38.4 | <0.001 |

LDL: low-density lipoprotein; HDL: high density lipoprotein; hypertension: systemic arterial hypertension

P-value determined by the chi-square test

**Table S2** – Prevalence of arterial hypertension (%) and systolic blood pressure (mean±SD; median and interquartile range), according to the genotypic frequencies for NOS3 rs1799983, TCF7L2 rs7903146, and IGFBP3 rs11977526 genes. Brazilian women descended from African descent, 2018.

| **Genotype** | **Genotype frequency (%)** | **Hypertension (%)** | **Systolic blood pressure^ǂ^** | |
| --- | --- | --- | --- | --- |
|  |  |  | **Mean*±*SD** | **Median (P25 – P75)** |
| *NOS3* **rs1799983^a^** |  |  |  |  |
| GG | 489 (47.9) | 24.5 | 123.8*±*17.9 | 120.5 (112.5 – 131.0) |
| GT | 261 (25.6) | 23.0 | 124.7*±*18.8 | 121.0 (112.5 – 132.0) |
| TT | 271 (26.5) | 52.0 | 132.2*±*21.9* | 129.0 (114.5 – 147.5)** |
|  |  |  | ***p<0.001 (Anova)*** | ***p=0.0001 (K-W)*** |
| *TCF7L2* **rs7903146^b^** |  |  |  |  |
| CC | 527 (51.6) | 27.9 | 125.44*±*19.21 | 122.0 (113.0 – 133.0) |
| CT | 304 (29.8) | 32.9 | 125.86*±*19.13 | 121.0 (113.5 – 134.8) |
| TT | 190 (18.6) | 38.9 | 129.13*±*21.15 | 124.5 (113.5 – 140.0) |
|  |  |  | ***p=0.0775 (Anova)*** | ***p=0.1118 (K-W)*** |
| ***IGFBP3* rs11977526^c^** |  |  |  |  |
| GG | 403 (39.5) | 24.1 | 123.49*±*18.04 | 120.0 (111.5 – 129.5) |
| AG | 447 (43.8) | 26.4 | 125.49*±*19.04 | 121.0 (112.5 – 134.0) |
| AA | 171 (16.7) | 62.0 | 134.78*±*22.13* | 131.0 (118.5 – 147.5)** |
|  |  |  | ***p<0.001 (Anova)*** | ***p=0.0001 (K-W)*** |

^a, b, c^ Hardy Weinberg Equilibrium:

*NOS3*, rs1799983: chi² =220.1159, *p=*0.606

[*TCF7L2*](https://www.ncbi.nlm.nih.gov/pubmed/30177026), rs7903146: chi²=112.3333, *p=*0.665

*IGFBP3*; rs11977526: chi² =6.009 *p=*0.613

**^ǂ^** One-sample Kolmogorov-Smirnov test against normal theoretical distribution: p<0.001 (systolic blood pressure distribution differs significantly from Gaussian distribution).

* Differs significantly from the others (*p<*0.01 according to the Bonferroni test).

K-W=Kruskal-Wallis test.

** Differs significantly from the others (*p<*0.01 according to the Dunnet test with Bonferroni correction).
